# ASK1 promotes uterine inflammation leading to pathological preterm birth

**DOI:** 10.1038/s41598-020-58653-9

**Published:** 2020-02-05

**Authors:** Midori Yoshikawa, Takayuki Iriyama, Kensuke Suzuki, Seisuke Sayama, Tetsushi Tsuruga, Keiichi Kumasawa, Takeshi Nagamatsu, Kengo Homma, Isao Naguro, Yutaka Osuga, Hidenori Ichijo, Tomoyuki Fujii

**Affiliations:** 1 Department of Obstetrics and Gynecology, Faculty of Medicine, The University of Tokyo, Tokyo, Japan; 2 Department of Developmental and Regenerative Biology, Medical Research Institute, Tokyo Medical and Dental University (TMDU), Tokyo, Japan; 3 Laboratory of Cell Signaling, Graduate School of Pharmaceutical Sciences, The University of Tokyo, Tokyo, Japan

**Keywords:** Reproductive disorders, Translational research

## Abstract

It is widely accepted that enhanced uterine inflammation associated with microbial infection is a main causative factor for preterm birth. However, little is known about the molecular basis by which inflammation is associated with preterm birth. Here, we demonstrate that apoptosis signal-regulating kinase 1 (ASK1), a member of the mitogen-activated protein 3-kinase family, facilitates inflammation-induced preterm birth and that inhibition of ASK1 activity is sufficient to suppress preterm birth. ASK1-deficient pregnant mice exhibited reduced incidence of lipopolysaccharide (LPS)-induced preterm birth. ASK1 was required for the induction of LPS-induced inflammatory responses related to preterm birth, including pro-inflammatory cytokine production in the uterus and peritoneal cavities. In addition, selective suppression of uterine ASK1 activity through a chemical genetic approach reduced the incidence of LPS-induced preterm birth. Moreover, translational studies with human choriodecidua demonstrated that ASK1 was required for LPS-induced activation of JNK and p38 and pro-inflammatory cytokine production. Our findings suggest that ASK1 activation is responsible for the induction of inflammation that leads to preterm birth and that the blockade of ASK1 signaling might be a promising therapeutic target for preventing preterm birth.

## Introduction

Preterm birth is a major health issue worldwide, occurring in 5–18% of all pregnancies, and is the leading cause of neonatal morbidity and mortality^1^. Excessive inflammation induced by an exaggerated immune response is known to trigger preterm birth and various other complications in pregnancy^2^. The key pathological mechanism underlying preterm birth is enhanced uterine inflammation associated with infection^3^. Bacterial infection ascending from the vagina to the uterus, and the subsequent progression to intrauterine inflammation are the most substantially confirmed causative factors for preterm birth^4,5^. Bacterial infection results in an increased production of pro-inflammatory cytokines, such as TNF-α, and triggers cervical ripening, uterine contraction, and fetal membrane rupture, all of which lead to preterm birth^6^. The above is supported not only by a number of clinical findings, but also by multiple pathophysiological findings from animal models in which preterm birth was triggered by administration of lipopolysaccharides (LPS) and other bacteria-derived substances^7^. However, despite intense research efforts to date, current strategies for treating preterm labor are limited to targeting bacterial infection and uterine contraction. These treatment options are insufficient with respect to improving perinatal outcomes, since increased myometrial contraction reflects the end stage of uterine inflammatory processes, which adversely affect the fetus^8^. Therefore, regarding therapeutics targeting preterm birth, there is an emerging interest in blocking the inflammatory cascade leading to excessive uterine inflammation, as it induces not only uterine contraction and subsequent preterm birth, but also several potential disorders in the fetus^9^. However, much remains unknown about the stress response systems that induce the enhanced detrimental inflammation leading to preterm labor and the details of the underlying molecular mechanisms.

Among a variety of stress-activated signaling systems, the stress-activated mitogen-activated protein kinase (MAPK) pathway converging on c-Jun N-terminal kinase (JNK) and p38 plays crucial roles in regulating cellular functions, including inflammation in response to various stressors^10^. Excessive activation of JNK and p38 is known to trigger pathologic inflammatory responses associated with enhanced production of pro-inflammatory cytokines in the development of various diseases, including cancers, autoimmune diseases, and microbial infections^11–13^. With respect to the pathogenesis of preterm birth, several recent studies using LPS-induced mouse models and human choriodecidua have suggested that the activation of the JNK and p38 pathways underlies the pathogenesis of inflammation-induced preterm birth^14,15^. However, findings implicating the involvement of stress-activated signaling systems in preterm birth are limited, and a mechanism detailing how JNK and p38 receive upstream signals and induce inflammatory responses that trigger preterm birth remains unknown.

The activities of JNK and p38 are tightly controlled by theirs upstream MAP kinase kinases and MAP kinase kinase kinases (MAP3Ks). Among MAP3Ks, apoptosis signal-regulating kinase 1 (ASK1) is accepted as a key player in the regulation of the activities of the JNK and p38 pathways, and plays pivotal roles in the pathogenesis of various diseases, including cancers, infections, and neurodegenerative diseases^16–18^. Intriguingly, ASK1 activation in macrophages and other inflammatory cells is crucial for Toll-like receptor 4 (TLR4)-mediated innate immunity responses triggered by LPS, which results in the production of pro-inflammatory cytokines; in fact, ASK1-deficient mice have demonstrated resistance to LPS-induced sepsis^19^. Moreover, because ASK1 is activated only under pathological conditions, it has drawn a great deal of attention as a therapeutic target^20^. However, very little has been determined regarding the involvement of ASK1 in pregnancy disorders, including preterm birth. In the present study, we demonstrate that ASK1 is crucial for promoting infection-induced uterine inflammation leading to preterm birth by regulating the JNK and p38 pathways, based on findings from an LPS-induced preterm birth model utilizing two independent types of ASK1-genetically-engineered mice, as well as *in vitro* studies using human choriodecidua, thus, implicating ASK1 as a potential therapeutic target for preterm birth.

## Results

### ASK1 deficiency suppresses LPS-induced preterm birth

**Figure 1:**
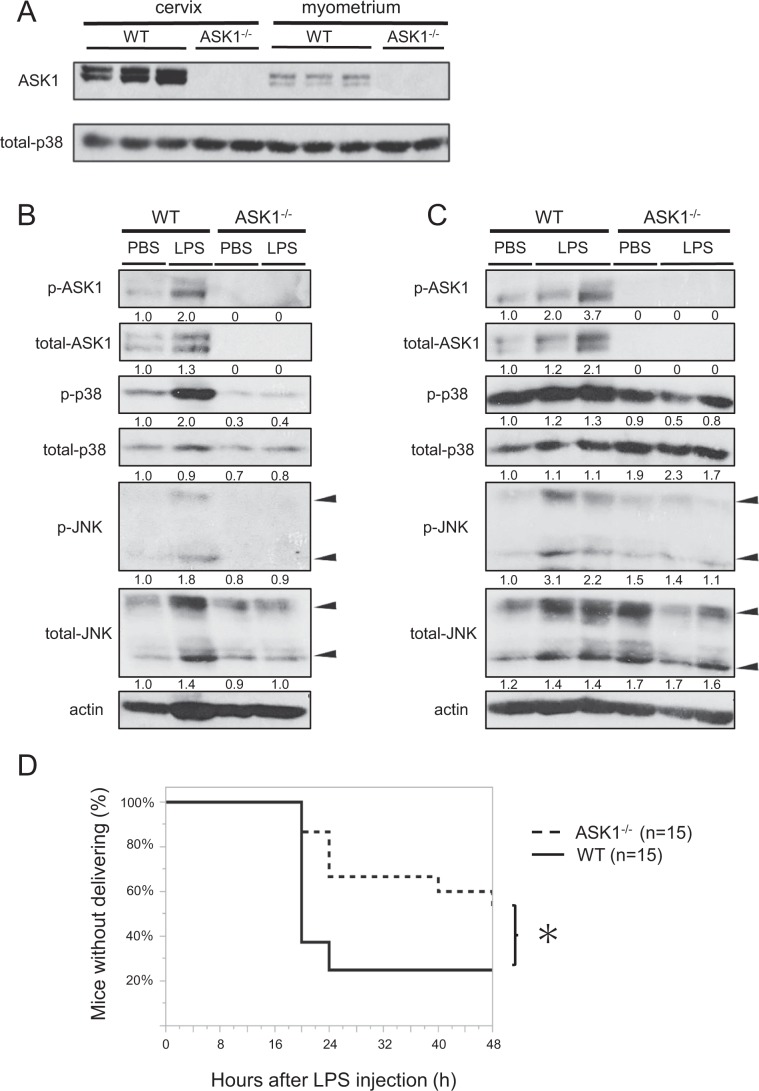
ASK1 deficiency suppresses LPS-induced preterm birth. (**A**) The expression of ASK1 in the cervix and myometrium of WT and ASK1^−/−^ pregnant mice detected by immunoblotting. Representative cropped images are presented. Uncropped images are shown in Fig. S1. (**B,C**) LPS (1.0 µg) or PBS was injected transvaginally into the cervix on embryonic day 15 of gestation. LPS-induced phosphorylation status of ASK1, JNK, and p38 in the cervix (**B**) and myometrium (**C**) was detected by immunoblotting at 8 hours following LPS or PBS injection into the cervix of WT and ASK1^−/−^ pregnant mice. These are representative images obtained from 3 to 5 mice per each group (**B**: n = 1 mouse in each group, **C**: n = 1 in PBS-treated groups and n = 2 mice in LPS-treated groups, included in these representative images). Numbers below the corresponding blot represent relative densitometric values of each blot normalized by actin. Uncropped images are shown in Fig. S2. (**D**) The incidence of preterm birth within 48 hours following LPS injection. Statistical analysis was conducted by Kaplan-Meier Method. *p < 0.05.

To examine the involvement of ASK1 in preterm birth, we initially assessed the expression of ASK1 in the uterus. ASK1 is reportedly expressed ubiquitously in mice, however, protein expression in the organs related to the female reproductive system remained unknown. Utilizing samples from ASK1-deficient (ASK1^−/−^) pregnant mice as negative controls, we confirmed that ASK1 protein is substantially expressed in the uterus, cervix, and myometrium (Fig. 1A). Then, to assess the roles of ASK1 in preterm birth, we used a preterm-birth mouse model induced by transvaginal injection of LPS into the cervix^21^, which mimics the pathological condition of chorioamnionitis resulting from bacterial infection ascending from the vagina up to the uterus, in wild-type mice and ASK1^−/−^ pregnant mice.

As a number of studies have previously reported, ASK1^−/−^ mice were viable and fertile, and displayed no apparent abnormal phenotypes in the female reproductive or other organs. In addition, no spontaneous preterm or post-term birth was induced in ASK1^−/−^ pregnant mice, and ASK1^−/−^ pups from ASK1^−/−^ pregnant mice were born and grew without any apparent abnormalities. First, the status of the uterine ASK1 signal transduction pathway, converging on stress-activated MAPKs, JNK, and p38, was examined by immunoblotting in the LPS-induced preterm birth model. Using a phospho-ASK1 antibody that specifically recognizes a critical threonine residue in ASK1^22^, which is the activating phosphorylation site, we found that cervical application of LPS enhanced the phosphorylation status of ASK1 both in the cervix and the myometrium of WT pregnant mice (Fig. 1B,C). In addition, we found that LPS-induced phosphorylation of JNK and p38 were suppressed in the cervix and myometrium of ASK1^−/−^ pregnant mice, compared with that in WT mice. These results suggested that uterine ASK1 is activated by LPS and that ASK1 is required for the activation of the JNK and p38 pathways in response to LPS in the uterus. Next, we examined the frequency of preterm birth and found that the incidence of delivery within 48 hours following LPS injection was significantly lower in ASK1^−/−^ pregnant mice than in WT mice (Fig. 1D). This result indicated that ASK1 functions to facilitate preterm birth induced by LPS.

### ASK1 facilitates LPS-induced inflammatory responses leading to preterm birth

**Figure 2:**
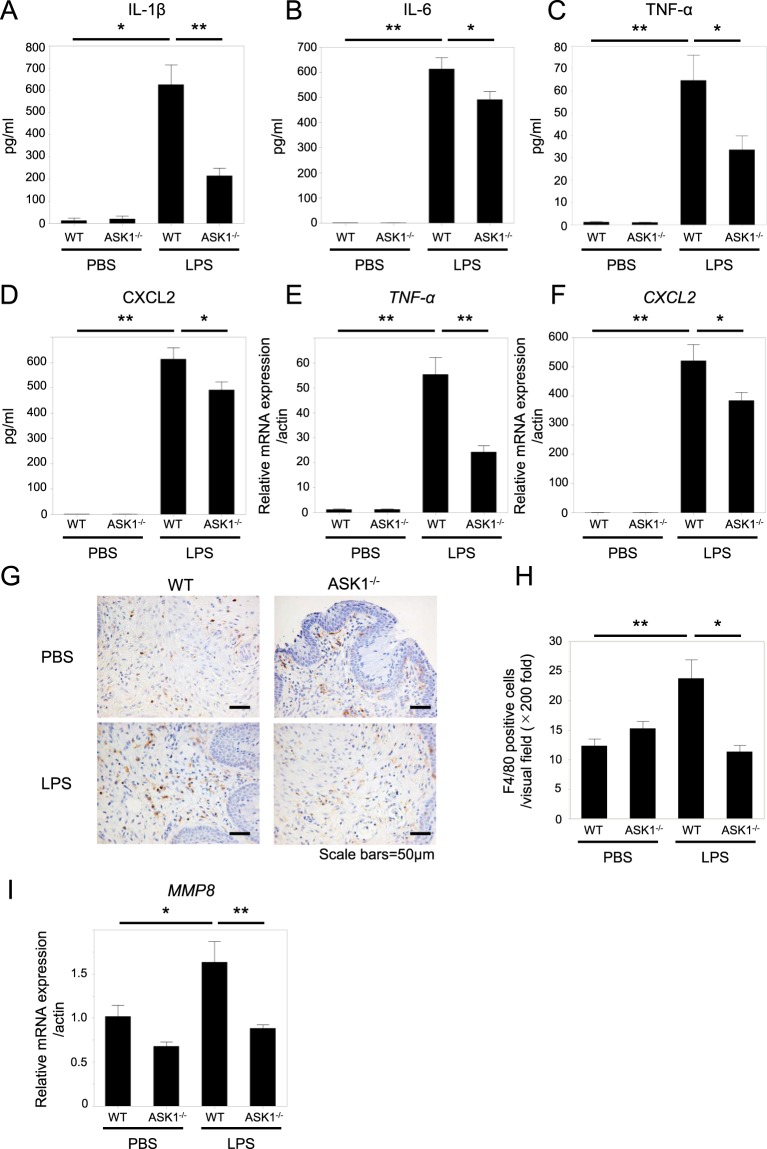
ASK1 facilitates LPS-induced inflammatory response leading to preterm birth. (**A–D**) The levels of IL-1 (**A**), IL-6 (**B**), TNF-α (**C**), and CXCL2 (**D**) in the peritoneal fluid taken 1 hour after LPS injection were measured by ELISA. (n = 7–14 mice in each group), (*p < 0.05, **p < 0.01). (**E,F**) mRNA expression levels of *TNF-*α (**E**) and *CXCL2* (**F**) in the myometrium at 1 hour after LPS injection were measured by real time RT-PCR. (n = 4–10 mice in each group), (*p < 0.05, **p < 0.01). (**G**) Representative images of immunohistochemical staining for F4/80, a maker for macrophages, in the cervix taken 8 hours after LPS injection. Scale bars = 50 μm. (**H**) The quantitation of the number of F4/80-positive cells in the stroma of cervix. The number of F4/80-positive cells was counted and averaged. (n = 3–4 in each group), (*p < 0.05, **p < 0.01). Cells counted as F4/80-positive in representative images presented in (**G**) are shown with arrows in Fig. S3. (**I**) *MMP8* mRNA expression levels in the cervix at 8 hours after LPS injection detected by real time RT-PCR. (n = 6–10 mice in each group), (*p < 0.05, **p < 0.01).

Preterm birth is characterized by locally enhanced inflammation in the uterus caused by microbial infection, including the production of pro-inflammatory cytokines and the infiltration of inflammatory cells into the uterus, which induces labor and cervical ripening, leading to the preterm birth^7^. LPS-induced activation of the JNK and p38 pathways is well known to facilitate the inflammatory response by also inducing cytokine expression as well^11^. Next, to assess the contribution of ASK1 to LPS-induced inflammation leading to preterm birth, we used ELISA to compare the levels of pro-inflammatory cytokines in the peritoneal fluid between WT and ASK1^−/−^ pregnant mice after LPS injection. The levels of IL-1β, IL-6, TNF-α, and the murine IL-8 homologue, CXCL2, in the peritoneal fluid were markedly increased following LPS treatment, and the elevated levels of these cytokines were significantly suppressed in ASK1^−/−^ pregnant mice compared with those in WT mice (Fig. 2A–D). Moreover, LPS-induced elevated levels of *TNF-α* and *CXCL2* in the myometrium were also significantly reduced in ASK1^−/−^ pregnant mice compared with WT mice (Fig. 2E,F). Among inflammatory cells amplifying the inflammation related to the pathogenesis of preterm birth, macrophages are the predominant subtype residing in the uterus^23^. Macrophages infiltrating the cervix are known to play critical roles in driving the inflammatory process that facilitates the cervical ripening mediated by the production of matrix metalloproteinases (MMPs)^24^. Therefore, we examined the state of macrophage infiltration in the cervix after LPS using immunohistochemical staining for F4/80, a marker for macrophages. LPS-induced cervical infiltration of macrophages with immunoreactivity for F4/80 was markedly visible in WT pregnant mice but was significantly less frequent in ASK1^−/−^ mice (Fig. 2G,H). Furthermore, we found that LPS-induced elevated levels of *MMP8*, a key player in the development of cervical ripening in preterm birth^25^, are significantly suppressed in the cervix of ASK1^−/−^ pregnant mice compared with WT mice (Fig. 2I). These results suggested that ASK1 plays crucial roles in the promotion of the inflammatory response that is closely associated with the development of LPS-induced preterm birth.

### Selective inhibition of ASK1 kinase activity elicits therapeutic effects on LPS-induced preterm birth

**Figure 3:**
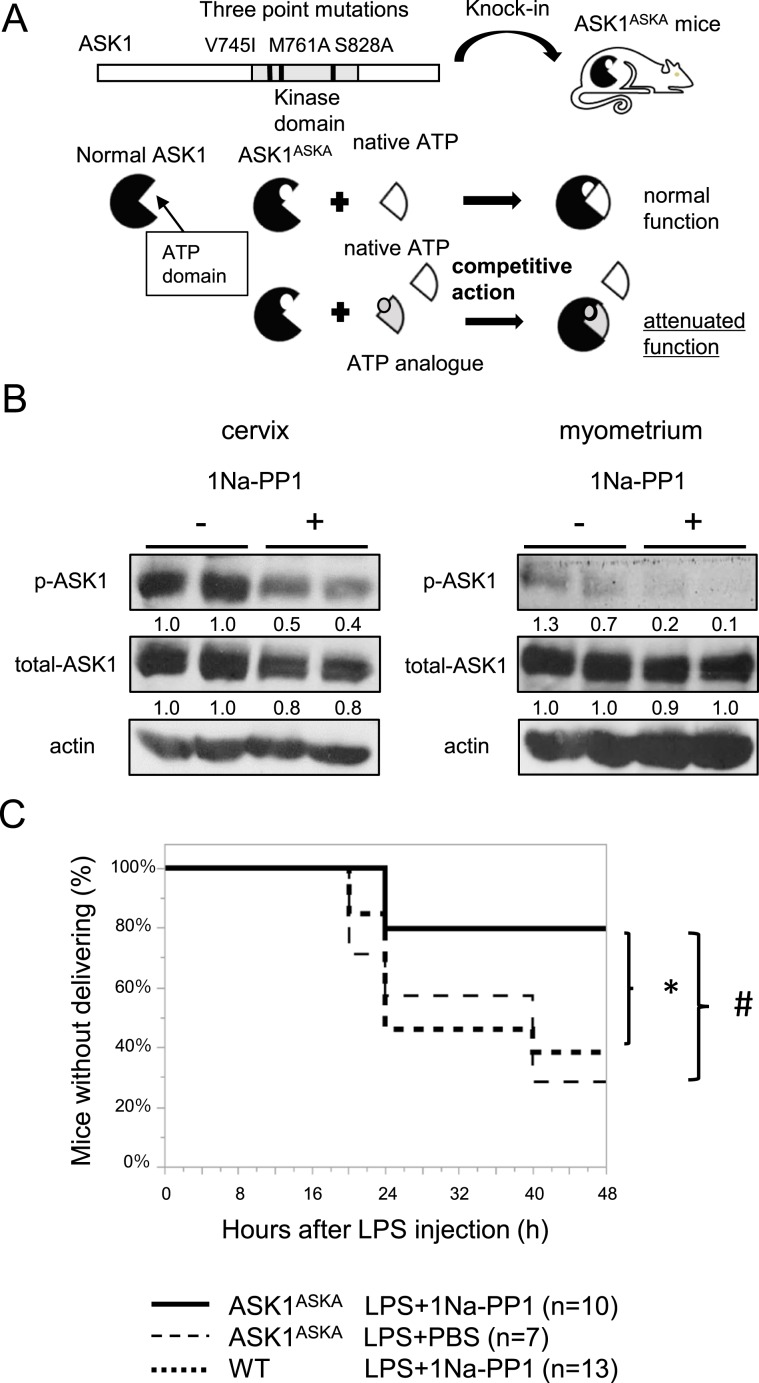
Selective pharmacological inhibition of ASK1 kinase activity suppresses the development of LPS-induced preterm birth. (**A**) Schematic diagram of an analogue sensitive kinase allele (ASKA) approach in which ASK1 activity can be pharmacologically inhibited. ASKA of ASK1 (ASK1^ASKA^) harbors triple mutations in the ATP-binding pocket of ASK1, which allows competitive inhibition by ATP analog without affecting the intrinsic kinase activity of ASK1 by native ATP. Knock-in mice harboring ASK1^ASKA^ were utilized for preterm birth experiment induced by LPS. (**B**) Cervical application of an ATP analog, 1Na-PP1, attenuated the activity of ASK1 in the cervix as well as myometrium. Phosphorylation status of ASK1 in cervices and myometrium at 2 hours after 1Na-PP1 treatment (952.2 µg/kg) was detected by immunoblotting using phospho-ASK1 antibody that can detect the activating phosphorylation of ASK1. Representative cropped images are presented (n = 2 mice in each group, included in these representative images). Numbers below the corresponding blot represent relative densitometric values of each blot normalized by actin. Uncropped images are shown in Fig. S4. (**C**) Selective suppression of uterine ASK1 activity by 1Na-PP1 treatment attenuated LPS-induced preterm birth in ASK1^ASKA^ pregnant mice. LPS (1.0 µg) was injected into the cervices of ASK1^ASKA^ pregnant mice or WT pregnant mice with or without 1Na-PP1 on embryonic day 15 of gestation. (*^,#^*p* < 0.05).

Our results that ASK1 deficiency suppressed LPS-induced preterm birth, as demonstrated using ASK1^−/−^ pregnant mice, raised the possibility that strategies to inhibit ASK1 kinase activity might possess therapeutic potential with respect to preterm birth. In order to investigate this possibility, we took a chemical genetic approach, termed analogue sensitive kinase allele (ASKA), in which ASK1 kinase activity can be pharmacologically controlled by the use of an ATP analogue^26^ (Fig. 3A). In this technology, an ASKA harbors mutations in the ATP binding pocket of its kinase domain, and while it can accommodate native ATP and function normally, its kinase activity is competitively blocked by an ATP analogue. We utilized knock-in mice harboring an ASKA of ASK1 (ASK1^ASKA^), in which selective pharmacological inhibition of ASK1 kinase activity can be achieved by the administration of an ATP analogue, 1Na-PP1. As previously reported^26^, the function of ASK1 in ASK1^ASKA^ mice in response to oxidative stress in the absence of 1Na-PP1 is comparable to that in WT mice. However, the oxidative stress-induced activation of ASK1 is robustly blocked by treatment with 1Na-PP1 in ASK1^ASKA^ mice, but not in WT mice. Then, utilizing ASK1^ASKA^ pregnant mice mated with ASK1^ASKA^ males, we performed transvaginal injection of LPS into the cervix and confirmed that the LPS-induced phosphorylation status of ASK1 in the cervix as well as the myometrium is substantially blocked by the application of 1Na-PP1 into the cervix in ASK1^ASKA^ pregnant mice (Fig. 3B). Intriguingly, similar to the preterm birth phenotype, transvaginal treatment with 1Na-PP1 into the cervix significantly reduced the incidence of preterm birth in ASK1^ASKA^ pregnant mice, compared with that in ASK1^ASKA^ pregnant mice without 1Na-PP1 treatment (Fig. 3C). As shown in Fig. 1D, in the absence of 1Na-PP1, the incidence of LPS-induced preterm birth in ASK1^ASKA^ pregnant mice was comparable to that in WT mice, suggesting that ASK1 functions normally in response to LPS in ASK1^ASKA^ pregnant mice. As there was no difference in preterm birth rates between WT mice with or without 1Na-PP1, the compound had no effect by itself on preterm birth induced by LPS. These results suggested that uterine ASK1 activity is responsible for LPS-induced preterm birth, and that a pharmacological approach to inhibit ASK1 kinase activity can elicit therapeutic effects.

### Suppression of ASK1 activity by the ASK1-specific inhibitor K811 reduced LPS-induced inflammation in human choriodecidua

**Figure 4:**
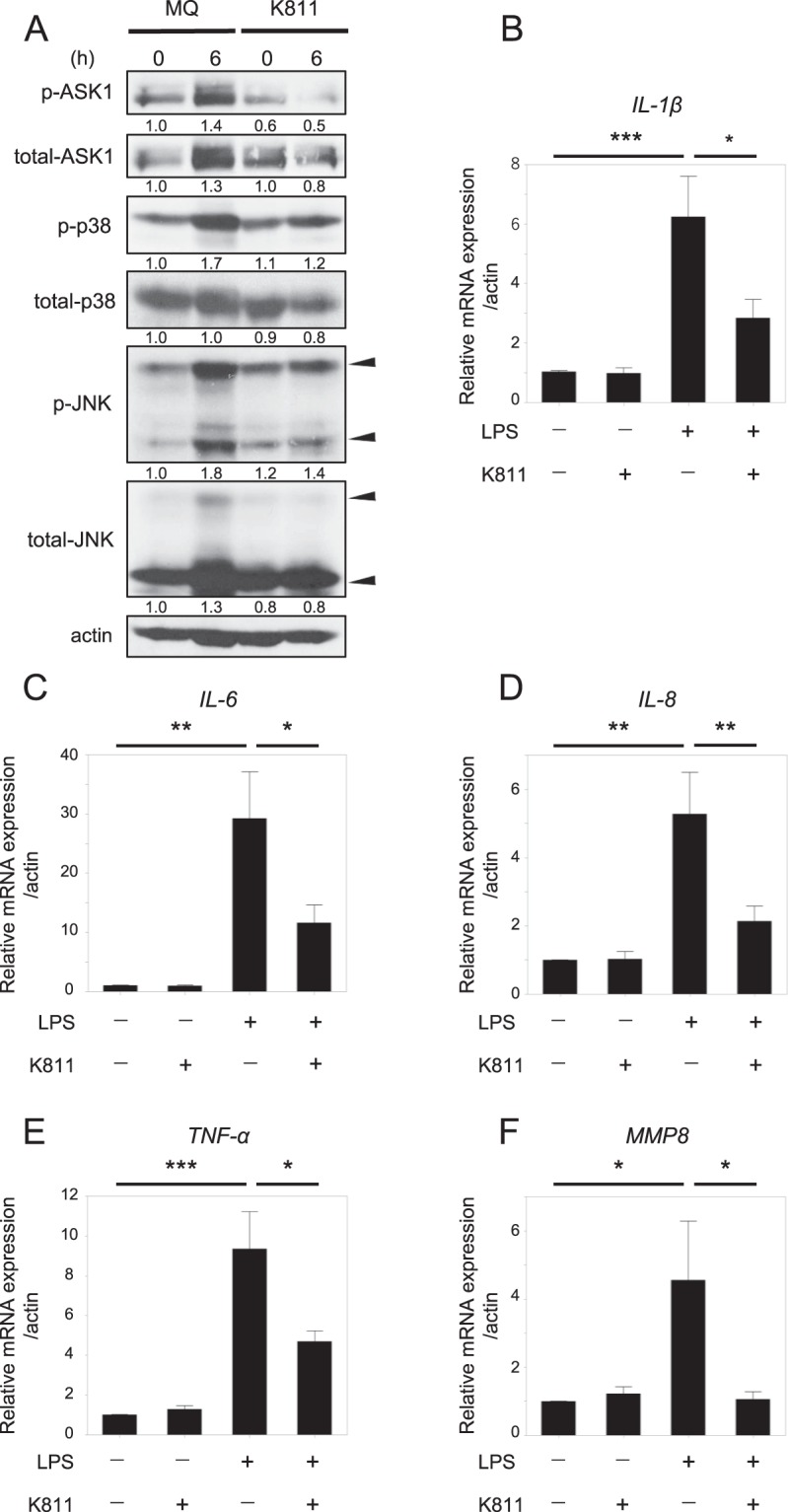
Suppression of ASK1 activity by ASK1 inhibitor reduces LPS-induced inflammatory responses in the human choriodecidua. Explant culture of choriodecidua was isolated from human term placentas from normal pregnancies. Explants were pretreated with or without 1 μM K811, a specific ASK1 inhibitor, for 1 hour and then treated with 0.5 μg/ml LPS. (**A**) Phosphorylation status of ASK1, JNK and p38 at the indicated periods after LPS stimulation was detected by immunoblotting. Representative cropped images are presented. Numbers below the corresponding blot represent relative densitometric values of each blot normalized by actin. Uncropped images are shown in Fig. S5. (**B–E**) K811 suppressed the induction of inflammatory cytokines, *IL-1*β (**B**), *IL-6* (**C**), *IL-8* (**D**), and *TNF-*α (**E**), by LPS treatment in the explant culture of human choriodecidua. mRNA expression levels of indicated cytokines at 6 hours after LPS treatment with or without K811 were determined by real time RT-PCR. (n = 9–10 experiments), (*p < 0.05, **p < 0.01). (**F**) *MMP8* expression induced by LPS was suppressed by K811 in the human choriodecidua. *MMP8* mRNA expression levels at 6 hours after LPS treatment with or without K811 was determined by real time RT-PCR. (n = 10 experiments), (*p < 0.05, **p < 0.01).

To extend these mouse findings to the human situation, we conducted *in vitro* studies using explant cultures of choriodecidua isolated from human term placentas from normal pregnancies. Choriodecidua, which infectious pathogens colonize in the initial stages of chorioamnionitis, plays a central role in triggering detrimental excessive inflammatory responses ascending to the intra-amniotic cavity by producing a number of pro-inflammatory cytokines^27^. Therefore, we explored the involvement of the ASK1-JNK and p38 pathways in LPS-induced responses in human choriodecidua by using a recently-developed ASK1-specific inhibitor, K811^28^. Immunoblotting analysis revealed that treatment with K811 blocked the LPS-induced phosphorylation status of ASK1 in human choriodecidua (Fig. 4A). Moreover, the phosphorylation of JNK and p38 induced by LPS in human choriodecidua was almost completely suppressed by K811 treatment. These results suggested that ASK1 is responsible for LPS-induced activation of the JNK and p38 pathways in human choriodecidua. Next, we conducted expression profiling of pro-inflammatory cytokines induced by LPS. mRNA expression levels of the pro-inflammatory cytokines, *IL-1β*, *IL-6*, *IL-8*, and *TNF-α*, were markedly increased in choriodecidua by LPS treatment, and the increased expression of these cytokines was significantly suppressed by the administration of K811 (Fig. 4B–E). We also found that LPS induced increased mRNA expression of *MMP8*, a key molecule whose overproduction is closely associated with the development of chorioamnionitis and preterm premature rupture of membranes^29^, in choriodecidua, and that K811 treatment significantly reduced the production of *MMP8* (Fig. 4F). These results indicated that ASK1 contributes to the induction of excessive inflammatory responses related to preterm birth in response to LPS in humans as well.

## Discussion

Utilizing two types of mice genetically engineered at ASK1 (ASK1-deficient mice and knock-in mice harboring an ASKA of ASK1), we revealed that ASK1 plays a critical role in the development of inflammation-associated preterm birth. Moreover, *in vitro* studies in human choriodecidua using an ASK1-specific inhibitor to suppress the activity of ASK1 demonstrated that ASK1 is responsible for LPS-induced inflammatory cytokine production related to preterm birth, by controlling the JNK and p38 pathways.

ASK1, a stress-activated MAP3K that regulates the activities of JNK and p38, plays key roles in stress responses under pathologic conditions^17^. A number of studies have so far reported that ASK1 is intimately associated with the progression of various diseases in which aberrant inflammatory conditions are involved^20^. However, the contribution of ASK1 to disorders of the female reproductive system, including obstetric complications, remained completely unknown. We initially found that ASK1 is robustly expressed in the uterus, pointing to the possibility that ASK1 functions in response to certain pathologic conditions during pregnancy. Then, considering the close involvement of ASK1 signaling pathways in innate immune response-induced inflammation^19^, the current study aimed to assess the roles of ASK1 in preterm birth. As pointed to by the abundant expression of ASK1 in the uterus, we found that uterine ASK1 is activated in response to cervical LPS injection and contributes to the activation of JNK and p38. Moreover, ASK1-deficient pregnant mice exhibited lower incidence of preterm birth induced by LPS, suggesting that ASK1 functions to facilitate inflammation-associated preterm birth. Thus, we report for the first time the involvement of ASK1 in the development of pregnancy complications, especially in inflammation-induced pathological preterm birth.

Excessive inflammation caused by microbial infection ascending into the cervix and uterus from the vagina is a key driving force in the development of preterm birth^6^. Increased inflammatory responses, characterized by enhanced production of pro-inflammatory cytokines and infiltration of leukocytes such as macrophages, are involved in every aspect of the preterm birth process, including induction of labor, cervical ripening, and rupture of the membrane. Several studies have reported the involvement of JNK or p38 in the pathogenesis of this infection-induced inflammatory process leading to preterm birth^14,15^. A recent study by Pirianov *et al*. reported that JNK activation contributes to the progression of LPS-induced preterm birth by facilitating uterine inflammation^14^. Intriguingly, this report also showed that the inhibition of JNK showed therapeutic effects, not only on preterm birth but also on excess inflammation-induced fetal brain damage. However, it has not been fully elucidated how the JNK/p38 pathways are involved in the enhanced inflammation leading to preterm birth. In the current study, we took advantage of a preterm birth model induced by transvaginal injection of LPS into the cervix, which mimics the pathological condition of chorioamnionitis, and found that LPS-induced production of IL-1β, IL-6, TNF-α, and the murine IL-8 homologue, CXCL2, was demonstrably reduced in ASK1^−/−^ mice compared to WT mice, as was the number of infiltrating uterine macrophages. All of these pro-inflammatory cytokines are reported to be locally elevated around the uterus after LPS injection in mice and to be involved in the progression to preterm birth^21^. In humans, IL-1β, IL-6, and TNF-α are increased in the myometrium, amniotic fluid, and decidua of pregnant women presenting with preterm birth, and are markers for the presence of infection^3^. Moreover, our findings using human choriodecidua revealed that ASK1 is required for LPS-induced MMP8 expression and production of IL-1β, IL-6, TNF-α, and IL-8. It has been reported that compared to the amnion, the choriodecidua is the major source of proinflammatory cytokines, including IL-1β, IL-6, IL-8, and TNF-α, which are closely associated with the development of preterm labor^27,30^. Shoji *et al*. reported that p38 activation promotes the production of these pro-inflammatory cytokines in human choriodecidua^15^, pointing to the significance of p38 in preterm birth and supporting our results. Thus, our results from ASK1-deficient mice combined with human choriodecidua strongly suggest that ASK1 plays crucial roles in the development of LPS-induced inflammatory responses leading to preterm birth.

Findings in ASK1^ASKA^ mice demonstrated that pharmacological inhibition of uterine ASK1 activity reduced the incidence of preterm birth induced by LPS. The ASKA technology is generally considered a powerful tool that allows the validation of specific responses to the inhibition of any protein kinase *in vivo* in knock-in animals^31^. The pharmacological application of an ATP analogue achieves selective competitive inhibition of the target kinase. Moreover, ASK1^ASKA^ enables the more precise investigation of ASK1 function without affecting protein expression of ASK1, since ASK1 deficiency induces the degradation of ASK2, a MAP3K molecule that is stabilized and functions in response to reactive oxygen species (ROS) only by forming a complex with ASK1^32^. Therefore, the findings from ASK1^ASKA^ mice showing that cervical treatment with 1Na-PP1 to suppress ASK1 activity reduced the incidence of preterm birth provided more solid *in vivo* evidence that uterine ASK1 activity is responsible for LPS-induced preterm birth, and that pharmacological inhibition of ASK1 kinase activity could be a novel therapeutic approach. As concerns human evidence, we demonstrated that a specific ASK1 inhibitor, K811, suppressed the LPS-induced activation of JNK and p38 and inflammatory responses related to preterm birth in human choriodecidua. Taken together, these findings strongly suggested that local uterine ASK1 inhibition could elicit anti-inflammatory therapeutic effects on inflammation-driven preterm birth. Several studies have reported that blockade of the JNK or p38 pathways have therapeutic potential for inflammation-induced preterm labor^14,15^. However, negative results from JNK and p38 inhibitors in clinical trials for various diseases, partly due to toxicity issues, indicate that the blockade of JNK or p38 is not desirable^13,33^. In contrast to the embryonic lethal phenotypes of mice deficient in p38 or JNK1/2, a number of studies have revealed that ASK1-deficient mice develop normally and display no abnormal phonotypes under normal physiological conditions^20^, indicating that ASK1 is not required for the maintenance of normal homeostatic mechanisms. ASK1 is a stress-responsive MAP3K, and it is activated and plays crucial roles only when cells are under stress. Thus, inhibition of ASK1 activity in preterm birth may aid in preventing ASK1-dependent activation of JNK and p38 in response to excessive ROS, which induces pathologic conditions, without interfering with the maintenance of homeostasis, including normal immune and anti-infection mechanisms. Theoretically, therapeutic strategies to inhibit ASK1 may carry low risks for side effects. Although further studies are needed to validate the safety of ASK1 inhibitors during pregnancy in animal models, our results suggest that ASK1 is an attractive target with respect to preterm birth, as it may enable the suppression of exaggerated inflammation cascades, which may lead to the improvement of perinatal outcomes.

In conclusion, our current study has provided valuable evidence from mouse and human models not only pointing to the significance of ASK1 signaling pathways in inflammation-associated preterm birth, but also establishing an innovative approach to inhibit preterm birth involving the anti-inflammatory effects of targeting ASK1.

## Methods

### Animals

All animal experiments were conducted according to the protocol approved by the Animal Research Committees of the Graduate School of Medicine, and of the Graduate School of Pharmaceutical Sciences of the University of Tokyo. All mice utilized in this study were of the C57BL/6J genetic background, and female mice at 8–12 weeks of age were used for all experiments. ASK1-deficient (Map3k5/ASK1^−/−^) mice and knock-in mice harboring an analogue-sensitive kinase allele (ASKA) of ASK1 (ASK1^ASKA^) mice were generated as described previously^26,34^. All mice were maintained at room temperature in a humidity-controlled room with a 12 hour-light/12 hour-dark cycle and had free access to food and water during the entire experimental period.

### Preterm birth model

The day of vaginal plug detection after the first day of mating was defined as day 0 of gestation. Lipopolysaccharide (LPS) derived from Escherichia coli (O111: B4; Sigma-Aldrich Japan, Tokyo, Japan) was injected transvaginally into the cervix on gestational day 15, as previously reported^21^. A volume of 200 μL of phosphate buffered saline (PBS) containing 1 µg of LPS was injected. In the control group, an equal volume of sterile PBS was used. ASK1^−/−^ pregnant mice mated with ASK1^−/−^ males or wild type (WT) pregnant mice mated with WT males were injected with LPS or PBS. Mice were observed at 16, 20, 24, 30, 40, and 48 hours after LPS or PBS injection. Mice were sacrificed at 48 hours after LPS injection. The time of delivery of the first fetus within 48 hours after LPS application was recorded and defined as preterm birth. To collect samples, mice were sacrificed at one and eight hours after LPS injection. The abdominal cavity was washed with 2 ml Milli Q water to obtain peritoneal fluid samples. To block ASK1 kinase activity in ASK1^ASKA^ pregnant mice, 4-Amino-1-tert-butyl-3-(19-naphthyl) pyrazolo [3,4-d] pyrimidine (1Na-PP1 952.2 µg/kg, Calbiochem, U.S.A.) was injected into the cervices of ASK1^ASKA^ pregnant mice mated with ASK1^ASKA^ male mice through the intracervical route. LPS (1.0 µg) was injected into the cervices of WT and ASK1^ASKA^ pregnant mice simultaneously with 1Na-PP1 on gestational day 15. Mice were sacrificed at two hours after LPS and 1Na-PP1 injection for sample collection.

### Immunoblotting

Tissues from mice and cultured human choriodecidua were lysed with lysis buffer (20 mM Tris-HCl pH 7.5, 150 mM NaCl, 10 mM EDTA pH 8.0, 1% Na-deoxycholate, 1% Triton X-100, 10% phosphatase inhibitor, 1 mM phenylmethylsulfonyl fluoride, 5 µg/ml Leupeptine). Supernatants collected by centrifugation were mixed with equal amounts of 2 × loading buffer (4% SDS, 100 mM Tris-HCl pH 8.8, 10% bromophenol blue, 36% glycerol, 10 mM dithiothreitol). The lysates were separated by SDS-PAGE and electroblotted onto polyvinylidene difluoride membrane (Millipore, U.S.A.). After blocking with 5% skim milk in TBS-T (250 mM Tris-HCl, 0.1% Tween20, 137 mM NaCl, 2.6 mM KCL), the membranes were probed with antibodies. The antibody/antigen complexes were detected using the ECL system (GE Healthcare, U.S.A.) as previously reported^34^. A polyclonal antibody to detect phospho-ASK1 (Thr845) was established as described previously^22,35^. Anti-phospho-JNK, anti-phospho-p38, anti-p38 antibodies (Cell Signaling Technology, U.S.A.), anti-JNK (Santa Cruz, U.S.A), anti-ASK1 (Abcam, U.S.A.), and anti-actin antibody (Sigma-Aldrich Japan, Japan) were purchased respectively. We quantified the densitometry of blot bands using imageJ software (National Institute of Health, U.S.A.).

### Quantification of mRNA expression

Total RNA was extracted from mouse tissues and human choriodecidua using Tissue Total RNA Kit (FAVORGEN, Taiwan). Total RNA was reverse-transcribed with qPCR RT Master Mix (TOYOBO, Japan). Quantitative PCR was performed with SYBR Green PCR Master Mix using the LightCycler 480 PCR machine (Roche Diagnostics K.K., Japan). The mRNA levels of mouse *TNF-α*, *CXCL2*, and *MMP8* were normalized to those of *β-actin* as internal control. The mRNA levels of human *IL-1β*, *IL-6*, *IL-8*, *TNF-α*, and *MMP8* were normalized to those of *β-actin* as internal control. Primer pair sets for each gene were purchased from Sigma-Aldrich, Japan. The primer pairs were the following: mouse β-actin: 5′-GCCTTCCTTCTTGGGTATGG-3′ and 5′-AGGTCTTTACGGATGTCAACG-3′; mouse TNF-α: 5′-TCTTCTGTCTACTGAACTTCGGGGTGA-3′ and 5′-GTGGTTTGCTACGACGTGGGCTA-3′; mouse CXCL2: 5′-TGTCAATGCCTGAAG ACCC-3′ and 5′-CTCTTTGGTTCTTCCGTTGAG-3′; mouse MMP8: 5′-CAACATTGCTTTCGTCTCAAGAG-3′ and 5′-GCATGGGCAAGGATTCCATT-3′; human β-actin: 5′-CATGTACGTTGCTATCCAGGC-3′ and 5′-CTCCTTAATGTCACGCACGAT-3′; human IL-1β: 5′-TACCTGTCCTGCGTGTTGAA-3′ and 5′-TCTTTGGGTAATTTTTGGGATCT-3′; human IL-6: 5′-GAACTCCTTCTCCACAACCG-3′ and 5′-TTTTCTGCCAGTGCCTCTTT-3′; human IL-8: 5′- ATGACTTCCAAGCTGGCCGT-3′ and 5′-TCCTTGGCAAAACTGCACCT-3′; human TNF-α: 5′-CCCGAGTGACAAGAATGTAG-3′ and 5′-TGAGGTACAGGCCCTCTGAT-3′; human MMP8: 5′-CCACTTTCAGAATGTTGAAGGGAAG-3′ and 5′-TCACGGAGGACAGGTAGAATGGA-3′.

### Measurement of inflammatory cytokine levels by ELISA

Levels of mouse IL-1β, IL-6, TNF-α, and CXCL2 in peritoneal fluid were measured by enzyme linked immunosorbent assay kit (Quantikine; R&D Systems, U.S.A.) according to the manufacturer’s protocol as described previously^36^.

### Immunohistochemistry

The tissues were fixed with 4% paraformaldehyde. The paraffin-embedded blocks and sections were obtained from Genostaff. (Genostaff Co., Ltd., Japan). Tissue sections were de-paraffinized with xylene and rehydrated through an ethanol series and PBS. For immunohistochemistry of F4/80, antigen retrieval was performed by enzyme treatment with Proteinase K. Endogenous peroxidase was blocked with 0.3% H_2_O_2_ in methanol for 30 min, followed by incubation with Protein Block (Genostaff) and the avidin/biotin blocking kit (Vector, U.S.A.). The sections were incubated with anti-F4/80 rat monoclonal antibody (BIO-RAD, Japan) at 4°C overnight. They were then incubated with biotin-conjugated rabbit anti-rat IgG (Vector) for 30 min at room temperature, followed by the addition of peroxidase conjugated streptavidin (Nichirei, Japan) for 5 min. Peroxidase activity was visualized by diaminobenzidine. The sections were counterstained with Mayer’s Hematoxylin (MUTO, Japan), dehydrated, and then mounted with Malinol (MUTO). For the quantification of F4/80 immunohistochemistry, the number of positively stained cells that have overlapping positive signals in the cell nucleus confirmed with the staining with hematoxylin was counted in 4 fields of randomized and blinded slides (200x magnification). Prior to immunostaining with F4/80, the specificity of the antibody was confirmed by using the spleen as a positive control and incubating biotin-conjugated rabbit anti-rat IgG as a secondary antibody without the primary antibody as a negative control.

### Explant culture of human choriodecidua

Experiments were conducted under the approval of the institutional review board of our institution (No. 11538-(3)), and informed consent was obtained from all pregnant women. Experiments were performed in accordance with the relevant guidelines and regulations of medical and health research involving human subjects. Human term membranes were collected from the placentas of cesarean sections performed at 37–38 weeks of gestation. Cases without maternal or fetal complications were selected for sample collection. Briefly, membranes isolated from the placenta were washed with sterile PBS and soaked in hyaluronidase (Nakalai tesque, Japan) for 30 minutes. A layer of choriodecidua was carefully scraped from amnion membranes. The tissues were cut into small pieces. Tissue explants (0.1 g) were placed in each well of a 24-well plate and cultured in DMEM/F12 (ThermoFisher SCIENTIFIC, Japan) containing 10% fetal bovine serum (FBS, ThermoFisher SCIENTIFIC) as described previously^15^. The ASK1 inhibitor K811 was developed and confirmed as a specific kinase inhibitor for ASK1 as reported previously^28^. Explants were pretreated with or without 1 μM K811 for 1 hour and then treated with 0.5 μg/ml LPS.

### Statistics

All data were expressed as the means ± standard error of the mean (SEM). Kaplan-Meier analysis was utilized to examine differences in rates of preterm births and a generalized Wilcoxon test was conducted to assess statistical significance. Differences between multiple groups were analyzed by one-way analysis of variance with Tukey’s HSD test. Statistical significance was set as *P* < 0.05 and analyzed by JMP (JMP, U.S.A.).

## Supplementary information

Supplementary Figure S1 to S5.
