# Supplementary material for: ASK1 promotes uterine inflammation leading to pathological preterm birth
Source: Sci Rep. 2020 Feb 5;10:1887. doi: 10.1038/s41598-020-58653-9 (PMC7002619; doi:10.1038/s41598-020-58653-9)

Supplementary Information

ASK1 promotes uterine inflammation leading to pathological preterm birth

Midori Yoshikawa[*1], Takayuki Iriyama[*1], Kensuke Suzuki[1], Seisuke Sayama[1], Tetsushi Tsuruga[1], Keiichi Kumasawa[1], Takeshi Nagamatsu[1], Kengo Homma[2], Isao Naguro[3], Yutaka Osuga[1], Hidenori Ichijo[3], and Tomoyuki Fujii[1]

[1]Department of Obstetrics and Gynecology, Faculty of Medicine, The University of Tokyo, Tokyo, Japan; [2]Department of Developmental and Regenerative Biology, Medical Research Institute, Tokyo Medical and Dental University (TMDU), Tokyo, Japan; [3]Laboratory of Cell Signaling, Graduate School of Pharmaceutical Sciences, The University of Tokyo, Japan

* These authors contributed equally to this work.

Correspondence: Takayuki Iriyama M.D., Ph.D.
Department of Obstetrics and Gynecology, Faculty of Medicine, University of Tokyo
7-3-1 Hongo, Bunkyo-ku, Tokyo, 113-8655, Japan
Tel; +81-03-3815-5411   Fax; +81-03-5800-6937   Email; iriyama-tky@umin.ac.jp

Supplementary Figure. S1

Figure S1 shows uncropped images. This figure corresponds to Fig.1A

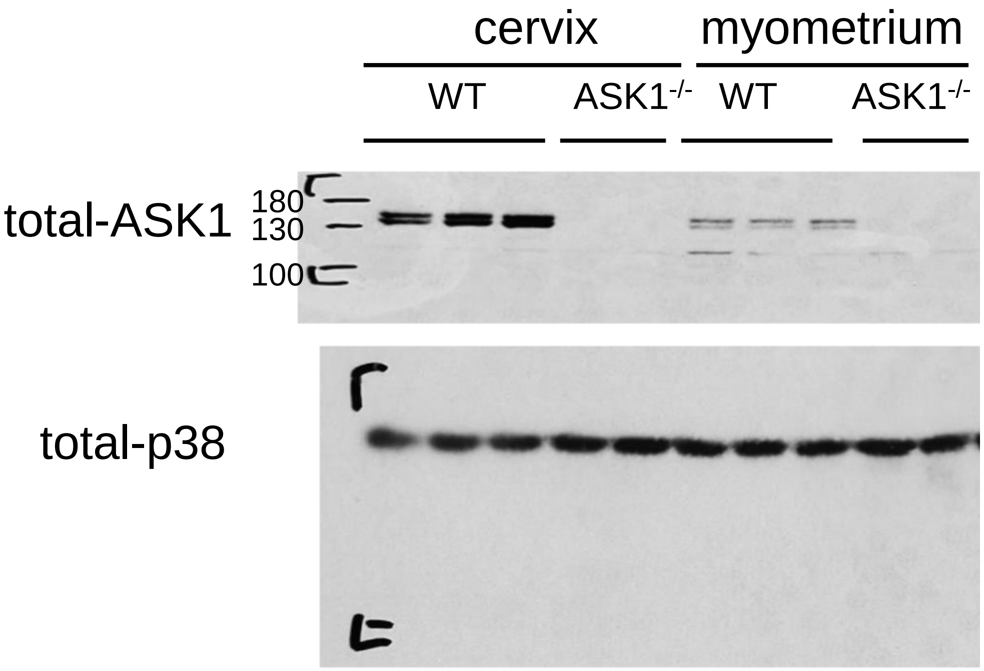

Supplementary Figure. S2

Figure S2 shows uncropped images. These figures correspond to Fig.1B and C.

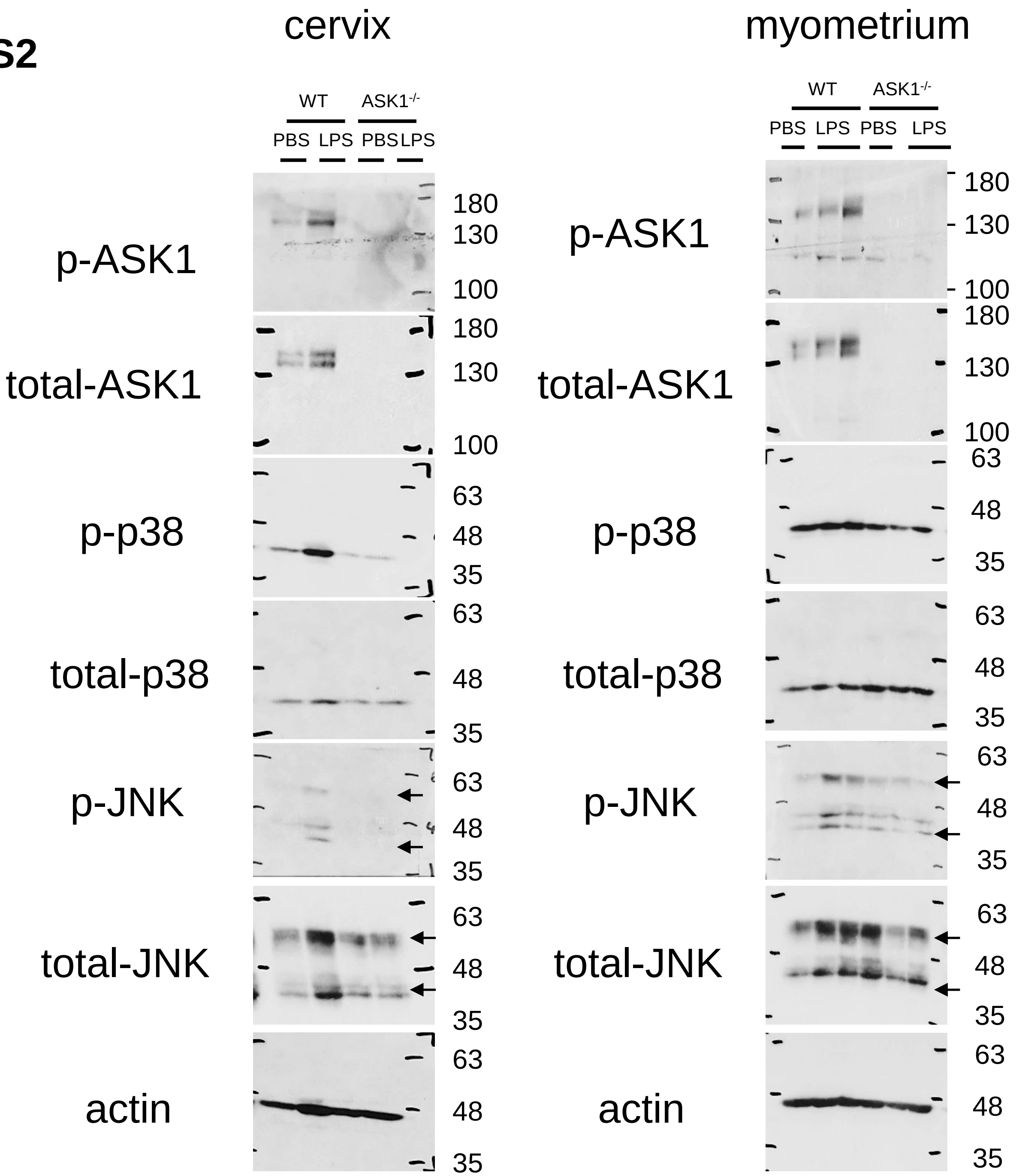

Supplementary Figure. S3

Cells counted as F4/80-positive in images presented in Fig. 2G are shown with arrows.

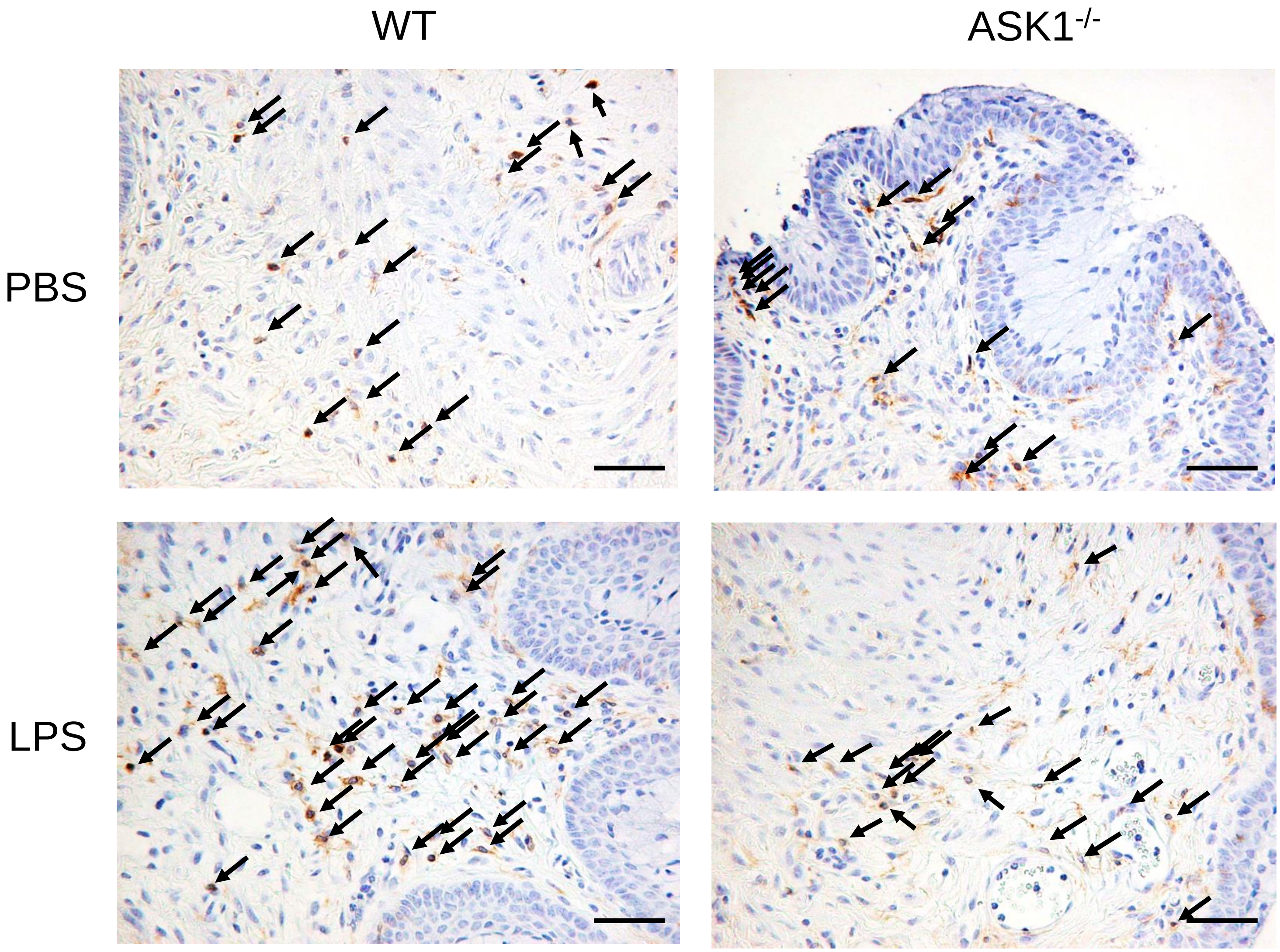

Scale bars=50μm

Supplementary Figure. S4

Figure S4 shows uncropped images. These figures correspond to Fig.3B.

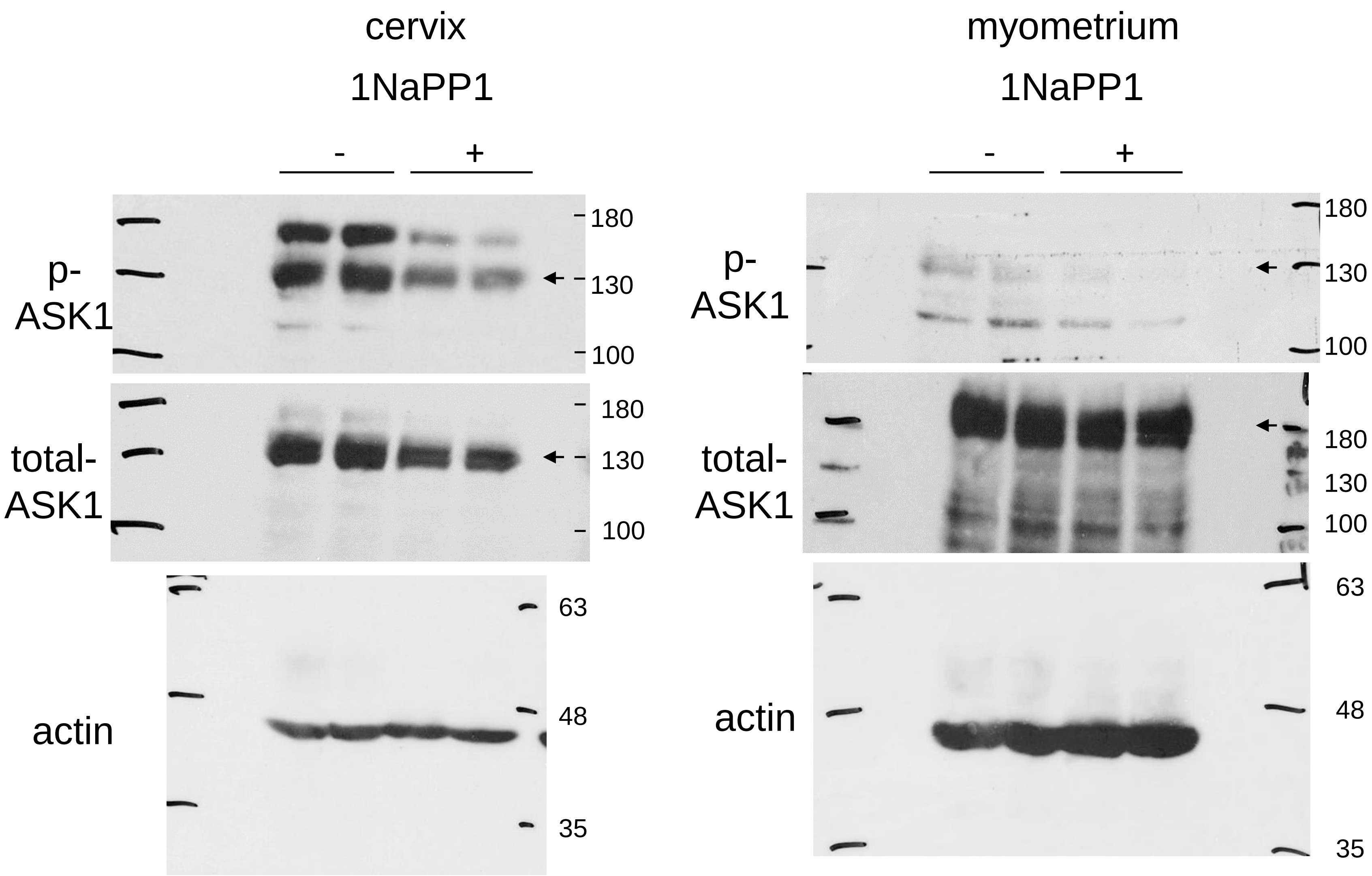

Supplementary Figure. S5

Figure S5 shows uncropped images. This figure corresponds to Fig.4A.

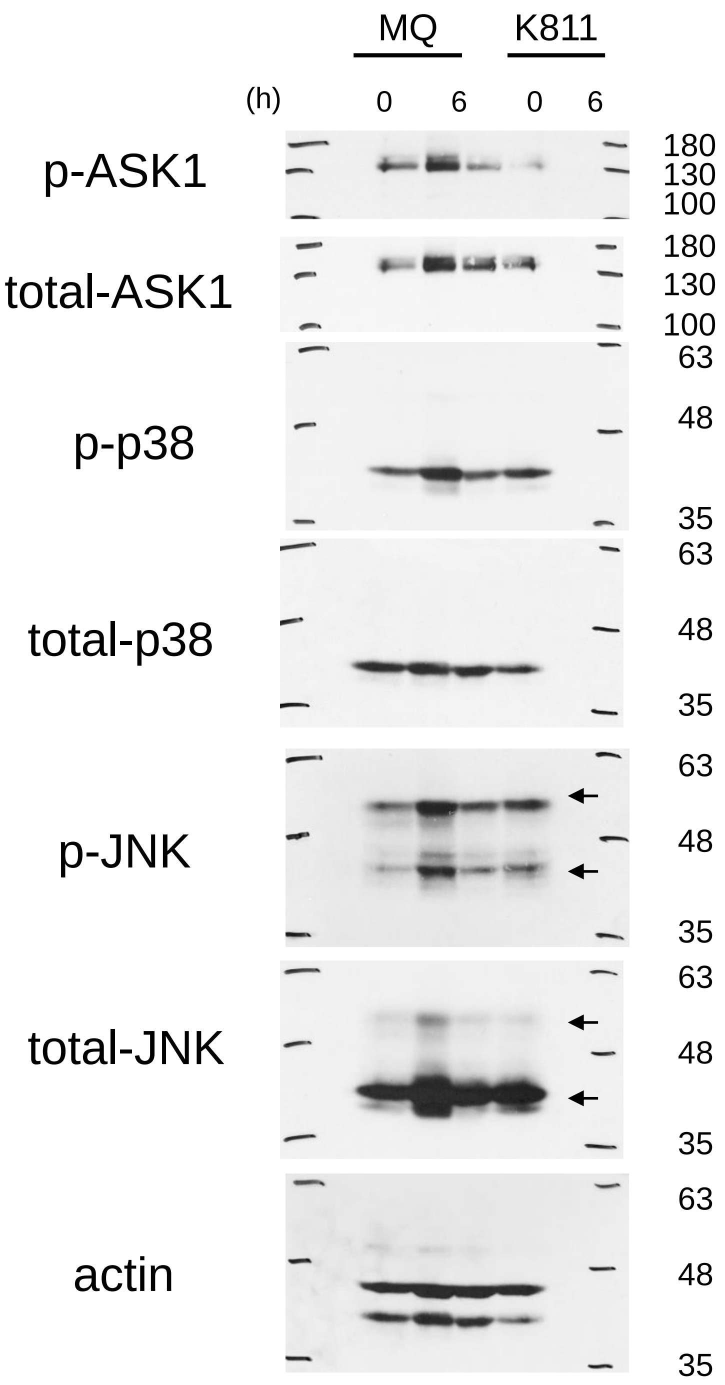

Supplement: Supplementary file 1 — Supplementary Figure S1 to S5. [file 41598_2020_58653_MOESM1_ESM.pdf]
